# Coactivation index of children with congenital upper limb reduction deficiencies before and after using a wrist-driven 3D printed partial hand prosthesis

**DOI:** 10.1186/s12984-018-0392-9

**Published:** 2018-06-08

**Authors:** Jorge M. Zuniga, Katsavelis Dimitrios, Jean L. Peck, Rakesh Srivastava, James E. Pierce, Drew R. Dudley, David A. Salazar, Keaton J. Young, Brian A. Knarr

**Affiliations:** 1 Department of Biomechanics, Biomechanics Research Building, 3D Printed Prosthetic, Orthotic & Assistive Devices, University of Nebraska, 6001 Dodge St, Omaha, NE 68182 USA; 2 Facultad de Ciencias de la Salud, Universidad Autónoma de Chile, Providencia, Chile; 3 Department of Exercise Science and Pre Health Professions, Creighton University, 2500 California Plaza, Omaha, NE 68178 USA; 4 CHI Health Creighton University Medical Center and an adjunct faculty at the Department of Occupational Therapy at Creighton University, Omaha, USA; 5 Innovative Prosthetics & Orthotics, Omaha, USA

**Keywords:** Additive manufacturing, Computer-aided design, Motor control, Reaching, Custom-made prostheses, Hand, Arm, Pediatric, Biomechanics

## Abstract

**Background:**

Co-contraction is the simultaneous activation of agonist and antagonist muscles that produces forces around a joint. It is unknown if the use of a wrist-driven 3D printed transitional prostheses has any influence on the neuromuscular motor control strategies of the affected hand of children with unilateral upper-limb reduction deficiencies. Thus, the purpose of the current investigation was to examine the coactivation index (CI) of children with congenital upper-limb reduction deficiencies before and after 6 months of using a wrist-driven 3D printed partial hand prosthesis.

**Methods:**

Electromyographic activity of wrist flexors and extensors (flexor carpi ulnaris and extensor digitorum) was recorded during maximal voluntary contraction of the affected and non-affected wrists. Co-contraction was calculated using the coactivation index and was expressed as percent activation of antagonist over agonist. Nine children (two girls and seven boys, 6 to 16 years of age) with congenital upper-limb deficiencies participated in this study and were fitted with a wrist-driven 3D printed prosthetic hand. From the nine children, five (two girls and three boys, 7 to 10 years of age) completed a second visit after using the wrist-driven 3D printed partial hand prosthesis for 6 months.

**Results:**

Separate two-way repeated measures ANOVAs were performed to analyze the coactivation index and strength data. There was a significant main effect for hand with the affected hand resulting in a higher coactivation index for flexion and extension than the non-affected hand. For wrist flexion there was a significant main effect for time indicating that the affected and non-affected hand had a significantly lower coactivation index after a period of 6 months.

**Conclusion:**

The use of a wrist-driven 3D printed hand prosthesis lowered the coactivation index by 70% in children with congenital upper limb reduction deficiencies. This reduction in coactivation and possible improvement in motor control strategies can potentially improve prosthetic rehabilitation outcomes.

## Introduction

Muscle co-contraction is the simultaneous activation of agonist and antagonist muscle groups around a joint and is measured using the coactivation index or CI [1–3]. Muscle coactivation is an essential and common motor control strategy for the execution of voluntary movement in healthy and clinical populations during locomotion [4, 5], maximal isometric contractions [6, 7], and upper-limb motor function [2, 3, 8, 9]. During upper-limb motor function, muscle coactivation has been shown to modulate the impedance and stability of a joint [2, 3, 8, 9]. However, the excessive coactivation can also impair motor performance due to the increased metabolic cost and the resulting lower joint net moments [7]. Thus, it is crucial to investigate the level of coactivation and muscle strength in clinical populations with upper-limb impairments [2, 3, 8, 9].

Children with congenital upper-limb reduction deficiencies face various issues and difficulties, but the extent of these difficulties depends on the location and level of the reduction [10]. These potential difficulties include abnormal development of motor skills, needing assistance with daily activities such as self-care, limitations with specific movements, sports, or daily activities. Specifically, children with partial hand reduction deficiencies have severe difficulties performing prehensile tasks with the involved limb [10]. Understanding the neuromuscular implications of prosthesis use is necessary to design and implement new prosthetic designs in addition to the use of clinical interventions to promote the motor development of children [11, 12] and increase prosthesis use longevity [13].

The use of 3D printed and standard transitional prostheses to restore and preserve strength and range of motion in children with upper-limb reduction deficiencies has been described by previous investigations [14–16]. In general, these transitional prostheses have shown to restore important clinical outcomes, such as gross dexterity [17], strength [14], and range of motion [16]. However, the motor control strategies of the developing neuromuscular system in children with congenital upper-limb reduction deficiencies have not been explored [12]. This information is crucial to understand the abnormal development of motor skills observed in children with congenital upper-limb reduction deficiencies and the role of prosthesis use [10].

Electric-powered units (i.e., myoelectric) and mechanical devices (i.e., body-powered) have improved to accommodate children’s functional needs, but their maintenance and replacement costs make access difficult for many families [18–20]. Voluntary-closing upper-limb prostheses are more suitable for children and could improve gross motor development [21, 22]. The Cyborg Beast 3D printed hand prosthesis was the first open-source design described in the scientific literature and it is characterized by using a voluntary-closing mechanism to produce grasping driven by wrist flexion [23]. This 3D printed hand prosthesis has been used to increase wrist range of motion [23] and gross dexterity [17] in children with congenital upper limb reduction deficiencies. Thus, the purpose of the current investigation was to examine the coactivation index of children with congenital-upper limb reduction deficiencies before and after using a wrist-driven 3D printed partial hand prosthesis. Specifically, we hypothesized that there will be a larger coactivation index and a lower strength value for the affected hand when compared to the non-affected hand prior to using a wrist-driven 3D printed transitional prosthesis. In addition, the coactivation index will decrease and strength will increase after using a wrist-driven 3D printed transitional prosthesis for a period of 6 months. This hypothesis was motivated by previous investigations that have reported increases of function [17], strength [14], and range of motion [16] after using transitional hand prostheses.

## Methods

### Experimental design

**Fig. 1:**
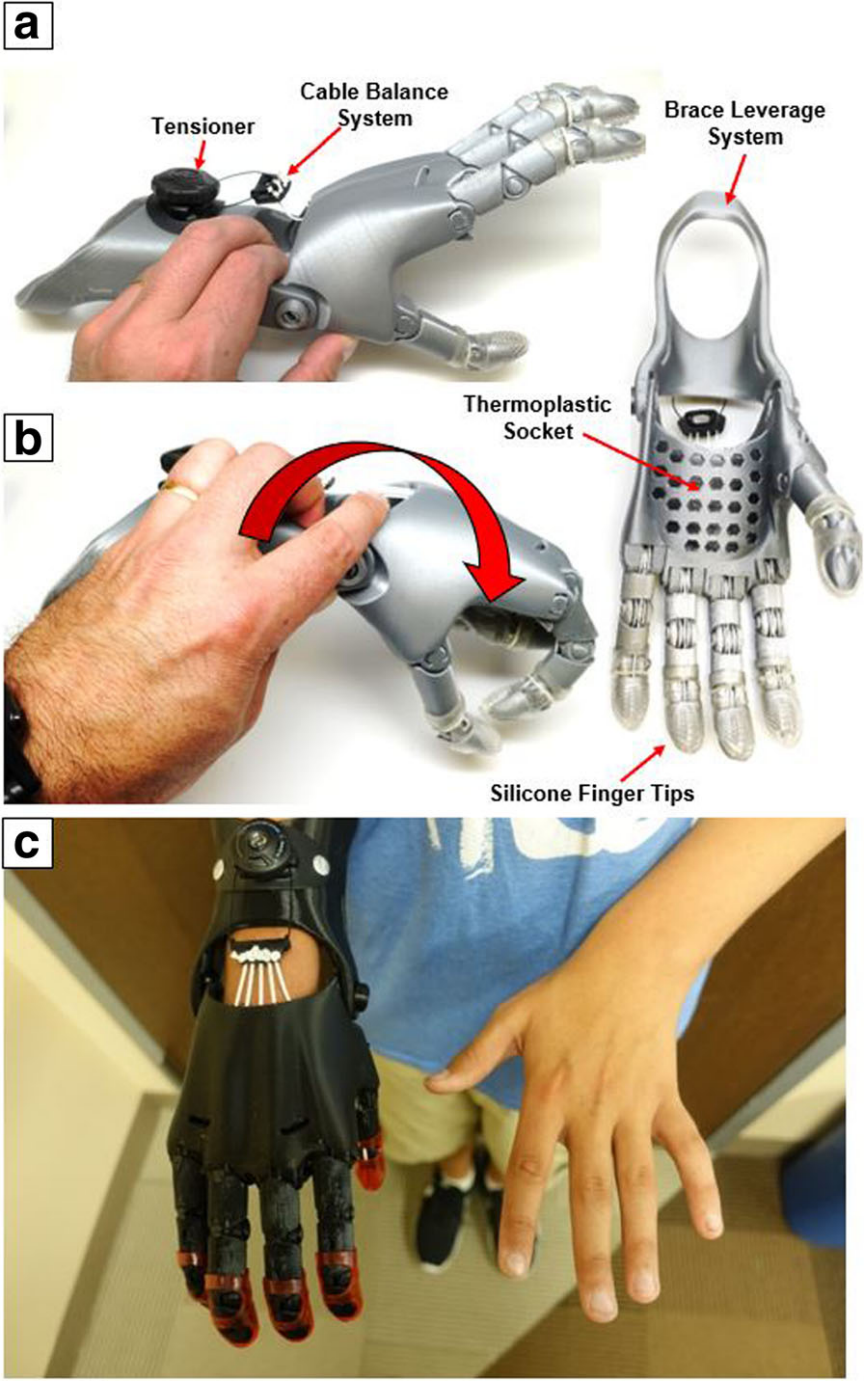
3D printed partial hand prosthesis (Cyborg Beast 2). **a** shows the hand prosthesis in the open position. Elastic cords placed inside the dorsal aspect of the fingers provide passive finger extension. **b** Finger flexion is driven by non-elastic cords along the palmar surface of each finger and is activated through 20–30° wrist flexion of the residual functional joint. The red arrow shows the direction of wrist flexion to close the fingers and produce a functional grasp. **c** 3D printed partial hand prosthesis fitted to subject 2

Electromyographic activity of wrist flexors and extensors (flexor carpi ulnaris and extensor digitorum) was recorded during maximal voluntary contraction of the affected and non-affected wrists. Co-contraction was calculated using the coactivation index (CI) and was expressed as percent activation of antagonist in comparison to the agonist. Measurements were performed before and after 6 months of using a wrist-driven 3D printed transitional hand prosthesis (Fig. 1).

### Participants

Inclusion criteria for all participants included boys and girls from 3 to 17 years of age with unilateral carpus upper-limb reductions, missing some or all fingers, and wrist range of motion of the affected wrist greater than 20°. Exclusion criteria included upper extremity injury within the past month and any medical conditions that would contraindicate the use of the transitional prosthesis, such as skin abrasions and musculoskeletal injuries. The study was approved by Creighton University Institutional Review Board.

Nine children (two girls and seven boys, 6 to 16 years of age) with congenital upper limb deficiencies participated in this study and were fitted with a wrist-driven 3D printed partial hand prosthesis (Table 1; Fig. 1). From the nine children, five (two girls and three boys, 7 to 10 years of age) completed a post baseline visit after using the wrist-driven 3D printed partial hand prosthesis for 6 months (Table 1).

**Table 1:** Characteristics of research participants (*n* = 9)

ID	Gender	Age (years)	Daily Prosthesis Use (hours)	Diagnosis	Ability to Pinch
1	M	7	3	Congenital deficiency right hand	No
2	M	10	2.5	Congenital deficiency right hand	No
3	M	16	1	Congenital deficiency left hand	No
4	M	9	3	Congenital deficiency left hand	No
5	M	8	2.5	Congenital deficiency left hand	No
6	F	6	4	Congenital deficiency left hand	No
7	F	7	2	Congenital deficiency left hand.	No
8	M	7	3	Congenital deficiency right hand	No
9	M	12	3	Congenital deficiency left hand	No
Mean		9.11	2.67		
SD		3.18	0.83		

All subjects completed a medical history questionnaire. All parents and children were informed about the study and parents signed a parental permission form. For children age 6 to 10 years of age, an assent was explained by the corresponding author and signed by the children and their parents. Detailed safety guidelines were given to the parents regarding the use and care of the prosthesis. Regarding prosthesis use, families were instructed to adopt a progressive schedule and monitor the children’s use of the wrist-driven prosthesis after 15 to 20 min looking for signs of onset of muscle soreness (i.e., muscle pain of the flexor muscles) and pressure points in the residual limb (i.e., redness in the skin) during the first 2 weeks of use. If any adverse event was encountered the families were instructed to discontinue use and contact any member of the research team. In addition, the families and children participating in this study completed a short survey. The survey was developed to estimate the impact of the prosthetic device on items related to quality of life, daily usage, and types of activities performed. The survey has not been statistically validated, but provides useful information related to daily usage.

Participants were asked to visit the laboratory on three occasions. During the first visit, a 3D scan of the upper-limbs was performed using a custom made epicyclical gearing system with a consumer grade optical 3D scanner (Sense 3D scanner, 3D systems Inc. Rock Hill, SC). Anthropometric measurements were also obtained to confirm the digital measurements obtained from the scan. After 3 weeks, research participants and their families returned to the laboratory for the prosthesis fitting and baseline testing. After 6 months of using the wrist-driven 3D printed partial hand prosthesis, participants visited the laboratory and repeated all assessments performed during the baseline testing. The maximal voluntary isometric contraction during wrist flexion and extension testing was performed by a certified occupational hand therapist. The prosthetic fitting was performed by a certified prosthetic and orthotic professional.

### Wrist-driven 3D-printed transitional partial hand prosthesis characteristics

A modified version of the 3D-printed transitional hand prosthesis named Cyborg Beast [24] (Fig. 1) was used in the study. The new version named Cyborg Beast 2 was designed using the modeling software Autodesk Fusion 360 (Fusion 360, Autodesk, Inc., San Rafael, CA, USA) and manufactured in the 3D Printed Prosthetic Orthotic & Assistive Devices Laboratory located in the Biomechanics Research Building of the University of Nebraska at Omaha. The 3D printers used for the manufacturing process included a combination of desktop and industrial 3D printers (Ultimaker 2, Ultimaker B.V., Geldermalsen, The Netherlands and Uprint SE Plus by Stratasys, Minnesota, USA).

The plastic pins to secure all the various components of the prosthesis, as well as the fingers and thumb were made of acrylonitrile butadiene styrene manufactured using an industrial 3D printer (Fig. 1). The palm, socket, forearm brace, and leveraging structure were made of polylactic acid which has properties similar to thermoplastic that facilitate post manufacturing adjustments. Elastic cords placed inside the dorsal aspect of the fingers provided passive finger extension. Finger flexion was driven by non-elastic cords along the palmar surface of each finger and was activated through 20–30 degrees of wrist flexion. The result was a composite fist (flexing the fingers towards the palm) for gross grasp. The finger and thumb were oriented in opposition to facilitate cylindrical grasp and tip pinch. A BOA dial tensioner system (Mid power reel M3, BOA Technology Inc., Denver, Colorado) was used to regulate the tension of the cables controlling the finger flexion. A brace leverage structure was included in the proximal aspect of the forearm to increase torque development and stability. A thermoplastic socket embedded in the palmar aspect of the hand prosthesis was added to facilitate fitting of the device. The hand prosthesis was customized to each child’s limb size and aesthetic requirements, such as colors and specific designs (Fig. 1).

### Isometric testing

Maximal voluntary isometric contraction during wrist flexion and extension was measured for both hands using a muscle testing dynamometer (microFET3, Hoggan Health Industries, West Jordan, UT). The investigator stabilized the applicator pad of the muscle testing dynamometer at the distal end of the affected hand and at a comparable location at the base of the palm of the non-affected hand. The participant’s arm and wrist were stabilized in a neutral position using a customized arm rest and Velcro straps with the elbow at an angle of 135° while seated in a chair. The subject was asked to flex and extend their wrist pushing on the pad of the muscle testing dynamometer as hard as possible for a period of 6 s. The subjects were given 2 min rest between contractions. Each measure was repeated three times for each motion and the average of the three measures was used for the analysis.

### EMG and coactivation index measurements

Two separate bipolar surface EMG electrode (circular 20 mm diameter, silver/silver chloride, Biopac Systems, Inc., Santa Barbara, CA) orientations (20 mm interelectrode distances) were placed over the flexor carpi ulnaris and extensor digitorum muscles of the affected and non-affected forearms. The parallel electrode orientation was placed in accordance with the recommendations from the SENIAM Project [25]. The skin at each electrode site was carefully abraded and cleaned with alcohol, and the impedance was less than 2000 Ω. The EMG signal from each electrode arrangement was amplified (gain: × 1000) using differential amplifiers (EMG 100, Biopac Systems, Inc., Santa Barbara, CA, bandwidth 1.0–500 Hz).

The raw EMG signals were digitized at 1000 Hz and stored for subsequent analyses. All signal processing was performed using custom programs written with LabVIEW programming software (Student Version 8.5.1, National Instruments, Austin TX). The EMG signals were bandpass filtered (fourth-order Butterworth) at 10–500 Hz, and the amplitude (microvolts root-mean-square, μVrms) was calculated for a 2.0 s time period corresponding to the middle 33% of the 6-s isometric muscle action.

Co-contraction was calculated by the coactivation index (CI) and was expressed as percent activation of antagonist over agonist [1].

$$ CI=\frac{EMG\ \mathrm{Antagonist}}{EMG\ \mathrm{Agonist}}\ast 100 $$

### Statistical analysis

A Shapiro-Wilk test was performed to analyze all data for normality with a C.I of 95%, with the null hypothesis of this test stating that the flexor and extensor strength of the affected and non-affected groups are normally distributed within the sample population. A Levene’s Test of Homogeneity was conducted to assess variance of the flexor coactivation index between groups. The null hypothesis of this test indicates that there is a large amount of variance between the affected and non-affected groups. This increased amount of variance would imply that the variability within each individual group is drastically different from the other (i.e. the variance of the affected group being significantly larger or smaller than the variance of the non-affected group).

Separate two-way repeated measures ANOVAs [2 × 2; hand (affected versus non-affected) x wrist motion (flexors and extensors)] were performed to analyze strength and coactivation index for 9 research subjects during the first visit. Separate two-way repeated measures ANOVAs [2 × 2; hand (affected versus non-affected) x time (before and after)] for flexion and extension were performed to analyze strength and coactivation index data for the first and second visit (before and after 6 months of prosthesis use). An alpha value of 0.05 was considered statistically significant for all comparisons.

## Results

**Fig. 2:**
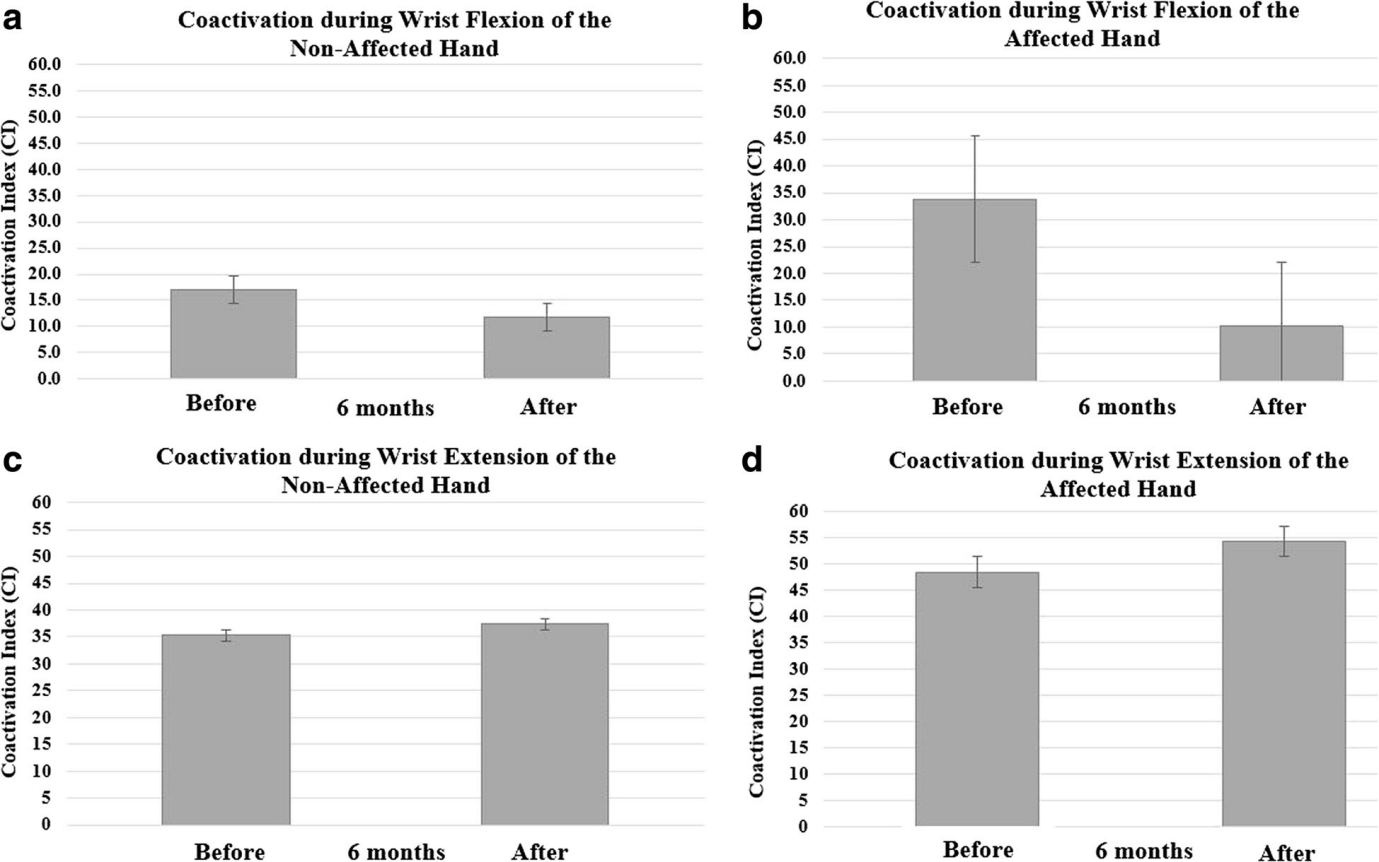
Coactivation Index (CI) during maximal voluntary isometric contraction of the wrist flexors of the non-affected hand (**a**) and affected hand (**b**) before and after 6 months of prosthesis use. CI during wrist extension of the non-affected hand (**c**) and affected hand (**d**) are also shown in this figure. Although no significant interactions were found in the current investigation, the affected hand exhibited a 70% reduction in the coactivation index after using the wrist-driven 3D printed hand prosthesis compared to a 30% reduction for the non-affected hand

**Fig. 3:**
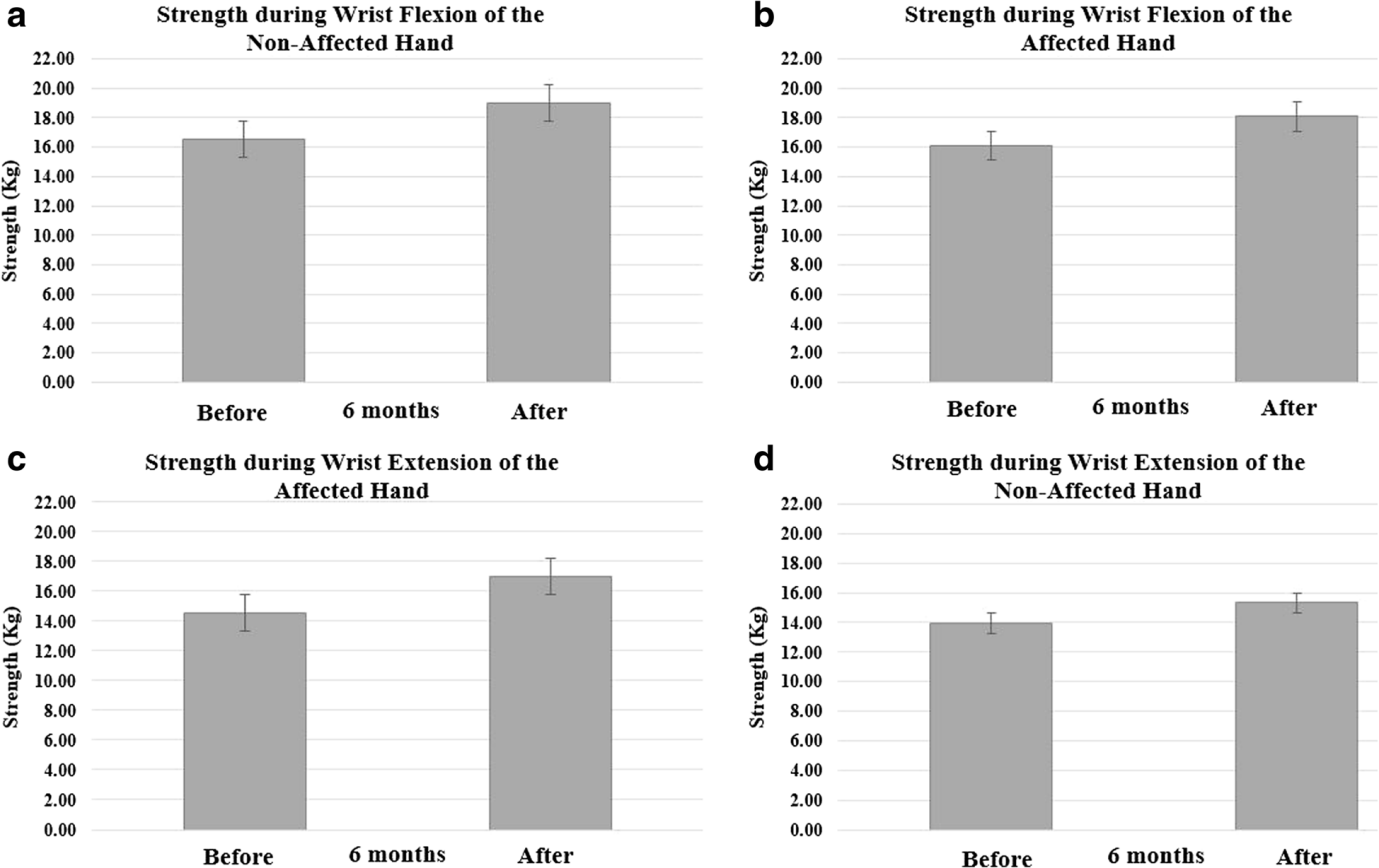
Strength (Kg) during maximal voluntary isometric contraction of the wrist flexors of the non-affected hand (**a**) and affected hand (**b**) before and after 6 months of prosthesis use. Strength (Kg) during wrist extension of the non-affected hand (**c**) and affected hand (**d**) are also shown in this figure

From the nine children participating in the study (Tables 1 and 2) only five (Table 3) completed the follow up visit after using the wrist-driven 3D printed transitional prosthesis for a period of 6 months (Fig. 2). The children that did not complete the follow up visit changed residency (subject 1, 5, and 9) or decided to discontinue using the hand prosthesis due to lack of interest (subject 3). Physical characteristics of the research participants are described in Table 1. Mean values (±SD) for strength and coactivation index measurements for the non-affected and affected hands (*n* = 9) are shown in Table 2. Mean (±SD) for strength and coactivation index measurements before and after 6 months of using a wrist-driven 3D printed hand prosthesis (*n* = 5) are shown in Table 3 (Fig. 3).

**Table 2:** Mean (±SD) strength measurements and coactivation index (CI) for the non-affected and affected hands (*n* = 9)

	Flexors Strength (Kg)		Extensors Strength (Kg)	
ID	Non-affected	Affected	Non-affected	Affected
1	16.96	23.8	12.96	14.96
2	20.4	20.46	18.73	19.2
3	26.9	27	19.6	24
4	15.7	12.3	15.8	14.6
5	23.93	22.33	18.06	15.9
6	4.20	5.20	4.40	3.0
7	14.8	25.2	16.5	16.5
8	27.53	17.43	17.36	16.36
9	10.8	8.3	8.9	7.73
M	17.91	18.00	14.70	14.69
SD	7.66	7.76	5.10	6.12
	Flexor Coactivation index (CI %)		Extensors Coactivation index (CI %)	
1	15.24	25.84	18.09	45.73
2	13.90	31.60	40.23	52.33
3	4.90	62.76	20.38	21.21
4	24.7	33.65	32.77	20.42
5	29.63	34.34	23.64	27.04
6	13.60	71.45	48.60	39.89
7	26.40	20.32	34.31	50.33
8	6.70	12.24	20.41	78.97
9	21.82	32.18	25.18	22.67
M	17.43	36.05	29.29	39.84
SD	8.70	19.13	10.38	19.41

**Table 3:** Mean (±SD) strength measurements and coactivation index (CI) before and after six months of using a wrist-driven 3D-printed hand prosthesis

	Flexors Strength (Kg)				Extensors Strength (Kg)			
	Non-affected		Affected		Non-affected		Affected	
ID	Before	After	Before	After	Before	After	Before	After
2	20.4	19.4	20.46	22.46	18.73	18.9	19.2	18.83
4	15.7	17.8	12.3	19.46	15.8	17.5	14.6	14.96
6	4.2	10	5.2	7.6	4.4	10.7	3.0	9.1
7	14.8	14.7	25.2	22	16.5	20.4	16.5	16.7
8	27.5	33.13	17.43	18.9	17.36	17.4	16.36	17
M	16.53	19.01	16.12	18.08	14.56	16.98	13.93	15.32
SD	8.54	8.67	7.70	6.06	5.78	3.72	6.33	3.74
	Flexor Coactivation index (CI %)				Extensors Coactivation index (CI %)			
2	13.9	4.6	31.6	13.9	40.2	48.3	52.3	85.5
4	24.7	6.4	33.7	24.7	32.8	46.2	20.4	47.3
6	13.6	29.7	71.5	13.6	48.6	21.4	39.9	79.4
7	26.4	6.9	20.3	26.4	34.3	26.6	50.3	29.8
8	6.7	11.7	12.2	6.7	20.4	44.4	79	29.6
M	17.1	11.9	33.9	10.3	35.3	37.4	48.4	54.3
SD	8.3	10.3	22.8	3.9	10.4	12.4	21.3	26.8

The Shapiro-Wilk test for the affected limb, before (*p* = 0.294) and after (*p* = 0.438) showed non-significant differences from the null hypothesis stating that these groups are normally distributed within the sample population. Similarly, trials of the non-affected limb for the first visit showed non-significant differences (*p* = 0.363) from the null hypothesis, while the trials for the second visit showed significance (*p* = 0.036). These results indicate that all conditions were normally distributed except the trial for the non-affected limb for the second visit.

The Levene’s Test of Homogeneity using a 95% confidence interval indicated that for all the trials tested there were no significant differences (*p* = 0.206) from the null hypothesis. These results suggest that the variances are homogenous between subject groups within the experiment.

### Affected versus non-affected upper-limbs

For strength during wrist flexion and extension there was a significant main effect for wrist motion [F (1, 8) = 12.78; *p* = 0.007, ηp^2^ = 0.62] indicating that flexors of the affected and non-affected hands produced more force than the extensors.

For coactivation index during wrist flexion and extension there was a significant main effect for hand [F (1, 8) = 12.5; *p* = 0.008, ηp^2^ = 0.61] indicating that the affected hand exhibited higher coactivation index for flexion and extension than the non-affected hand. No other main effects or interactions were significant.

### Before and after 6 months of prosthetic use

For strength during wrist extension there was a significant main effect for hand [F (1, 4) = 8.23; *p* = 0.04, ηp^2^ = 0.68] indicating that the non-affected hand produced more force than the affected hand.

For coactivation during wrist flexion there was a significant main effect for time [F (1, 4) = 9.9; *p* = 0.03, ηp^2^ = 0.71] indicating that the affected and non-affected hand had a significantly lower coactivation index after a period of 6 months (Fig. 2). No other main effects or interactions were significant. The affected hand exhibited a 70% reduction in the coactivation index after using the wrist-driven 3D printed hand prosthesis compared to a 30% reduction for the non-affected hand.

All nine families and children participating in this study completed a short survey. After 6 months of using the 3D printed hand prosthesis, children and their families reported using the hand for 2.7 ± 0.83 h a day (Table 1). Furthermore, children reported using the prosthetic hand “just for fun” (*n* = 8), for “activities at home” (*n* = 4), to “play” (*n* = 9), for “school activities” (*n* = 3), and to perform “sports” (*n* = 3).

## Discussion

The main findings of the present investigation showed that the affected hand of children with unilateral upper-limb reductions had a significantly higher coactivation index for wrist flexion and extension at baseline. Furthermore, the use of a wrist-driven (by flexion) 3D printed hand prosthesis lowered the coactivation index during wrist flexion by 70% compared to a 30% reduction for the non-affected hand. Although no significant interactions were found in the current investigation, a 70% reduction in the coactivation index after using a 3D printed hand prosthesis (Table 3 and Fig. 2b) may be clinically relevant. Previous investigations have examined coactivation during isometric muscle actions in clinical populations of children and adolescents with cerebral palsy [7, 26] as well as adults with a wide range of neurological pathologies [6]. In general, these studies have found a greater coactivation of the antagonist compared to the agonist during submaximal and maximal isometric muscle contractions [6, 7, 26]. For individuals experiencing acquired limb amputations, the studies of muscle coactivation have been explored as an option for myoelectric control of upper-limb prostheses [27] or to examine the state of coactivation patterns of the residual limb musculature of trans-tibial amputees [5]. Seyedali et al., [5] reported that the coactivation index of the residual limb of lower limb amputees was significantly greater than the intact limb and a control group during gait [5]. However, children with congenital (*n* = 3) and acquired (*n* = 2) trans-tibial limb reduction produced significantly smaller coactivation during gait when compared to a control group suggesting poor knee stability [4].

Little is known about the patterns of coactivation in the residual upper-limb musculature of children with congenital upper-limb reductions. It has been reported that coactivation in the upper-limbs is an effective motor control strategy that is primarily observed when an individual needs increased joint stability or improved movement accuracy while learning a new motor task [2]. However, due to the inherent inefficiency and increased metabolic cost, the excessive coactivation seen in clinical populations can also impair motor performance [7]. The results from the present investigation do not only indicate that the affected side of children with congenital upper-limb reductions showed a significantly higher coactivation index during wrist flexion and extension (Table 2), but also that the coactivation index was reduced by 70% when using a wrist-driven 3D printed hand prosthesis (CI before = 33.86 ± 22.78% versus CI after = 10.3 ± 3.9%). The higher coactivation index found in the affected hand of children with congenital upper-limb reductions agrees with previous investigations performed in children with cerebral palsy [26] and adult stroke patients [3]. Steenbergen et al., [26] examined upper-limb function of the less-affected side in young adolescents with congenital hemiparesis and found these participants showed weak positive correlations between agonist (triceps) activity and elbow amplitude, suggesting that deficient agonist rather than antagonist innervation was responsible for the decreased elbow involvement. Hammond et al., [3] examined the coactivation in the hemiparetic forearm of stroke patients and age-sex matched control subjects during maximal voluntary isometric wrist flexion and extension. The authors found that the coactivation index was significantly greater for the stroke patients than controls. It was concluded that both agonist recruitment and antagonist inhibition are impaired in the hemiparetic arm [3]. The main difference between patients with cerebral palsy or stroke and the population in the current study is the compromised central nervous system. Children with congenital upper-limb reductions have no apparent dysfunction at the central nervous system. There is some evidence, however, showing minor neurological dysfunctions in children with congenital below-elbow deficiency [11]. A longitudinal study of four children with upper-limb reductions found that three children presented minor neurological dysfunction and two children presented a complex form associated with developmental coordination disorder [11]. These findings are partially supported by another study that found that after a 24-year follow-up, individuals with unilateral upper-limb reductions report difficulties performing unilateral and bimanual motor tasks resulting in lower performance in sports and musical activities indicating a minor motor delay [28]. Changes in coactivation can provide information related to the motor control strategies of the central and peripheral nervous system [2, 9, 29], which can be used to improve prosthetic rehabilitation outcomes [11]. Studies performed in able-bodied participants [2, 9, 29] have shown that the brain and cerebellar cortex uses “internal models” of the body to control arm movement [29]. In the context of upper-limb function, internal models are neural representations of how the hand and arm would respond to a neural command [29]. It is possible that the reduction in coactivation during flexion rather than extension after using a wrist-driven 3D printed transitional prostheses was a reflection of the development of a new “internal model” to control this device.

Recent investigations have reported that cortical reorganization is continuously modified in healthy adults in response to activity, behavior, and skill acquisition [30]. Similarly, cortical reorganization also occurs after injury to the central nervous system (stroke) and peripheral injury (amputations) [30]. For congenital reductions, however, previous investigations have suggested that children with congenital unilateral upper-limb reductions may lack representation of the missing part of the limb in the cerebral cortex [12, 13, 31, 32] and that prewired movement representations of a limb need the experience of movement to be expressed within the primary motor cortex [12]. Consequently, the child may have a limited number of “motor repertoire” for the affected upper-limb, limiting motor function. [25] Thus, it is conceivable that the higher coactivation index found in the affected hand compared to the non-affected hand of children with congenital upper-limb reductions may be a result of limited number of “motor repertoire” restricting mobility of wrist and promoting the use of the affected hand only for object stabilization during bimanual activities [33].

The decreased coactivation index during flexion found in the affected hand after 6 months of using a wrist-driven 3D printed hand prosthesis agrees with previous investigations that have found that coactivation in the upper-limbs decreases over the course of learning a novel motor task [2, 3, 8, 9]. It has been proposed that coactivation may be a strategy that is used by the central nervous system early in learning a novel task to improve control compensating for the lack of motor commands [2, 8]. Following this rationale, it is possible that after 6 months of using the wrist-driven 3D printed partial hand prosthesis coactivation was reduced as learning took place and neural representation of this task was formed to improve control [2, 8]. The reduction in coactivation after a period of using the device was reflected during the maximal voluntary isometric flexion of the wrist of the affected hand (Table 2 and Fig. 2b). This reduced coactivation may be clinically relevant as it provides a potential assessment to examine the state of coactivation patterns of the residual limb musculature of children with congenital upper limb reductions with possible applications to myoelectric control of upper-limb prostheses.

The peripheral mechanisms responsible for the reduction of coactivation in our research subjects after using a wrist-driven 3D printed partial hand prosthesis can be explained by the repeated flexion of the residual wrist of the affected hand of the children participating in the present investigation. Specifically, the wrist-driven 3D printed partial hand prosthesis may have increased the stimulus to the highly innervated scapholunate interosseous ligament of the wrist producing desensitization [34, 35]. The scapholunate interosseous ligament was intact in the affected limb of our research subjects demonstrated by the presence of wrist mobility and functional range of motion (20° to 30°). As shown by Hagert et al., 2009 [35] coactivation of wrist flexors and extensors occurred after stimulation of the scapholunate interosseous ligament. Furthermore, it has been shown that coactivation after rehabilitation regime including eccentric, concentric, or isometric wrist exercises improves patient ability to more effectively activate flexors or extensors controlling the coactivation pattern to produce a balanced wrist motion [34]. Thus, it can be speculated that the simple action of flexing the wrist repeatedly to close the finger of the wrist-driven 3D printed hand prosthesis may have produced a desensitization of the scapholunate interosseous ligament contributing to the reduction in the coactivation index observed after the prolonged use of this device [34, 35]. More evidence is needed to assess the contributions of the scapholunate interosseous ligament and rehabilitation exercises to changes in coactivation of the wrist muscles of children with congenital partial hand reduction deficiencies.

The potential limitations of the present investigation are related to the lack of an age-matched control group, a small number of children participating in the study and their wide range of ages (6 to 16 years of age). All these factors may have contributed to our lack of significant interactions. The current study did not include an age-matched control group to assess the typical development of muscle activation and strength in age-matched children over the time span of the study. However, the contralateral arm was used as a control, as suggested and described in previous investigations [8, 9]. A sample size of nine children (2 girls and 7 boys) made it difficult to group research participants by age and gender. For example, the difference in age shown in Table 1, the resulting inter-subject variability in strength shown in Table 2, and the small sample size (*n* = 5) of children that completed a visit after using the wrist-driven 3D printed hand prosthesis for a period of 6 months may have contributed to the non-significant changes in strength found in the current study (Table 2). The reduction in coactivation of the forearm flexors with no changes in strength after using a 3D printed hand prosthesis is consistent with findings in typically developing children showing no correlations between isometric muscle strength and coactivation of hamstrings during maximal isometric muscle actions of the knee extensors [6]. The lack of correlation between muscle strength and coactivation of lower limb muscles of pediatric subjects illustrates the complexity of the motor control strategies of the developing neuromuscular system [6]. Furthermore, the applicator pad of the muscle testing dynamometer in the present investigation was positioned at the distal end of the affected hand and at a comparable location at the base of the palm of the non-affected hand. Therefore, it is possible that errors in the positioning of the applicator pad during strength testing of the non-affected hand may have changed the moment arm of the wrist joint affecting the torque development and artificially decreasing the strength values reported for the non-affected hand of subject 1 and 7 in Table 2.

Future investigations should examine the influence of using upper-limb prostheses in the brain activation patterns of the motor cortex and the neural plasticity changes in children experiencing congenital and acquired limb loss. In addition, a comprehensive electromyographic examination of the muscles controlling the upper-limb prosthesis will provide critical information about the state of the coactivation patterns of the remaining muscle structure of children with congenital upper-limb reductions. Lastly, establishing a dose-response of daily prosthesis use and neuromuscular improvements will provide crucial information to clinicians and patients to reach specific clinical outcomes enhancing current prosthetic rehabilitation programs.

## Conclusion

The present investigation showed that the affected hand compared to the non-affected hand of children with unilateral upper-limb reductions had a larger coactivation index for wrist flexion and extension at baseline. More importantly, the use of a wrist-driven 3D printed hand prosthesis lowered the coactivation index during wrist flexion by 70% compared to a 30% reduction for the non-affected hand in children with congenital upper limb reduction deficiencies. This reduction in coactivation and possible improvement in motor control strategies can potentially improve prosthetic rehabilitation outcomes for children with congenital upper limb reduction deficiencies.

## Abbreviations

3D: Three dimensional
ANOVAs: Analysis of variance
CI: Coactivation Index
EMG: Electromyography
SENIAM: Surface EMG for non-invasive assessment of muscles
